# Corynebacterium Striatum Native Valve Endocarditis

**DOI:** 10.12669/pjms.42.(11AASC).15796

**Published:** 2026-04

**Authors:** Sara Ahmed, Ibtesam Ishrat, Bilal Memon, Rabbiya Mohatarrem

**Affiliations:** 1 Sara Ahmed, Department of Medicine, Aga Khan University, Karachi, Pakistan; 2 Ibtesam Ishrat, Department of Medicine, Aga Khan University, Karachi, Pakistan; 3 Bilal Memon, Department of Medicine, Aga Khan University, Karachi, Pakistan; 4 Rabbiya Mohatarrem, Department of Medicine, Aga Khan University, Karachi, Pakistan

**Keywords:** Aortic root abscess, *Corynebacterium striatum*, Infective endocarditis

## Abstract

*Corynebacterium* species are traditionally regarded as harmless commensals, but nondiphtherial strains, such as *C. striatum*, are now recognised to cause clinically invasive infections, including infective endocarditis. We describe a case of a 25 years old male with a bicuspid aortic valve who presented with persistent fever and was found to have *C. striatum* bacteremia. Echocardiography revealed multiple vegetations on the aortic valve with aortic regurgitation and an aortic root abscess. Despite being on vancomycin and having therapeutic levels, his fever persisted, so he was switched to linezolid. He developed congestive heart failure, requiring urgent aortic valve replacement with abscess drainage. He recovered well postoperatively and remained stable at one-month follow-up. This case emphasizes the significance of recognizing *C. striatum* as a rare cause of native valve endocarditis, particularly in young adults with structural heart disease. It highlights the importance of species-level identification, as *C. striatum* is often multidrug-resistant and has an aggressive course, which directly influences antibiotic selection and timely surgical intervention, thereby improving outcomes.

## INTRODUCTION

*Corynebacterium* species are gram-positive, non-spore-forming bacilli that are usually found on the skin and mucous membranes. Historically, this genus was most notable for *Corynebacterium diphtheriae*, the pathogen responsible for diphtheria. However, in recent years, non-diphtherial species—often referred to as diphtheroids have emerged as clinically significant.^1^ Once dismissed as mere contaminants, now these organisms are recognised as pathogens that can cause a spectrum of localised and systemic infections, particularly in immunocompromised patients or those with indwelling medical devices.^2^ Clinically important nondiphtherial species, such as *C. jeikeium*, *C. urealyticum*, and *C*. *striatum*, have been implicated in bacteremia, endocarditis, and urinary tract infections, highlighting the growing importance of accurate identification and targeted antimicrobial therapy in patient management.^1^,^2^

Infective endocarditis (IE) caused by nondiphtherial *Corynebacterium* species is uncommon but increasingly recognised, particularly in patients with predisposing conditions such as underlying valvular disease, prosthetic heart valves, intracardiac, nosocomial devices, or immunocompromised states.^3^ It is estimated to cause 9% of early prosthetic valve endocarditis (PVE) and 4% of late PVE, but only 0.2–0.4% of native valve endocarditis (NVE).^4^ While prosthetic valve endocarditis accounts for most cases, native valve endocarditis caused by *C. striatum*, especially in young adults, is rare.^5^ We report a case of infective endocarditis complicated by aortic root abscess caused by *C. striatum* in a young adult with a bicuspid aortic valve necessitating surgical aortic valve replacement.

## CASE PRESENTATION

A 25 years old male nurse from Peshawar with a history of treated pulmonary tuberculosis and a known bicuspid aortic valve presented with a 10 days history of high-grade, intermittent fever accompanied by chills, rigors, and two episodes of watery, non-bloody diarrhea. He denied cough, urinary symptoms, recent travel, or sick contacts. One week prior, he had been admitted elsewhere with a diagnosis of dengue fever based on a positive dengue NS1 antigen and treated with intravenous fluids; however, he left against medical advice due to persistent fever before presentation to our facility.

On examination, he looked pale and was febrile with a temperature of 104°F. Cardiovascular examination revealed a diastolic murmur best heard at the aortic area and widened pulse pressure. The rest of the systemic examination was unremarkable. Laboratory investigations showed mild leukopenia (total leukocyte count 4.1×10^9^/L), relative lymphocytosis (53.6%), and thrombocytopenia (91×10^9^/L). Serum electrolytes revealed hyponatremia (131 mmol/L), while kidney and liver function tests were normal. Dengue serology was positive for IgM antibodies.

Considering his recent hospitalization and the potential for healthcare-associated infection, he was empirically started on vancomycin and meropenem as he was a healthcare provider. Two sets of blood cultures were sent, which subsequently grew *Corynebacterium* species; however, urine and sputum cultures remained sterile. An initial transthoracic echocardiogram revealed a bicuspid aortic valve with vegetation, mild aortic regurgitation, moderate mitral regurgitation, and a small echogenic density suggestive of vegetation. After 48 hours of antibiotic therapy, his repeat blood cultures were negative. Later identification by MALDI-TOF MS confirmed *Corynebacterium striatum* sensitive to gentamicin, vancomycin, and linezolid and resistant to meropenem. Vancomycin trough levels were monitored, and despite therapeutic levels being achieved, he remained febrile, so he was switched to linezolid.

**Fig.1:**
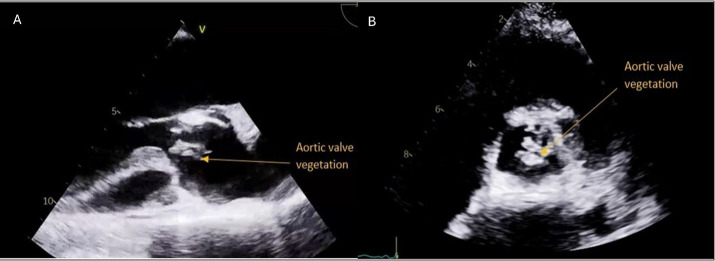
Transthoracic echocardiogram, A-parasternal long axis view, B-parasternal short axis view showing echogenic mass on the aortic valve (arrows), representative of aortic valve vegetation.

During his hospital stay, he developed worsening heart failure requiring non-invasive ventilation. Further evaluation with transesophageal echocardiography revealed multiple vegetations on the bicuspid aortic valve, leaflet perforation, severe eccentric aortic regurgitation, and a posteriorly located aortic root abscess communicating with the aortic lumen, and moderate-to-severe mitral regurgitation (Fig.1). However, no vegetation was identified on the remaining cardiac valves, including the mitral, tricuspid, and pulmonary valves. At the same time, both ventricles maintained normal systolic function. Given the extent of valvular destruction with aortic root abscess, urgent surgical intervention was performed.

The patient underwent metallic aortic valve replacement, including removal of the vegetation and drainage of the aortic root abscess. Intraoperative tissue cultures were negative. No surgical intervention was performed on the mitral valve; mitral regurgitation was managed conservatively. Postoperatively, he was prescribed warfarin with monitoring of INR levels, which were maintained. He showed steady clinical improvement and was subsequently discharged. He completed a six-week course of antibiotics. At the one-month follow-up, he remained clinically stable, INR was maintained and he returned to his hometown. Approximately two weeks after the last follow-up, however, he died from an intracerebral haemorrhage.

## DISCUSSION

This case highlights the significance of recognizing *C. striatum* as a rare cause of native valve infective endocarditis, specifically in young adults with an underlying bicuspid aortic valve. Although *Corynebacterium* species are often considered contaminants in routine blood cultures, a retrospective population-based analysis of 335 episodes of *Corynebacterium* bacteremia found that only 30 cases (8.8%) represented true infection.^6^ However, in patients with echocardiographic evidence of endocarditis, such isolates should not be regarded as contaminants and must be considered clinically significant. In this patient, repeated positive blood cultures, a clinically compatible picture, along with echocardiographic evidence of valve destruction, were consistent with endocarditis. This case demonstrates the need for clinicians to interpret the findings of blood culture in the clinical context and risk factors.

Most reports of *C. striatum* endocarditis involve prosthetic valves, intravascular devices, immunosuppression, and extended hospital stays.^3^,^4^,^5^,^7^ In this patient, the only predisposing factor for developing *Corynebacterium* endocarditis was the presence of a bicuspid aortic valve and peripheral intravenous catheters used for fluid administration for dengue. *Corynebacterium* colonizes skin and mucous membranes, and a breach in the integrity of skin can lead to bacteremia in high-risk patients and in those with indwelling lines.^7^

Nondiphtheroidal *Corynebacterium* species vary in pathogenicity and antimicrobial susceptibility, so species-level identification is essential. Matrix-Assisted Laser Desorption/ Ionization Time-of-Flight Mass Spectrometry (MALDI-TOF MS) has become a reliable method for fast and precise identification of *Corynebacterium* species, aiding therapeutic decision-making.^2^ Although bactericidal agents such as β-lactams or vancomycin are typically preferred in bloodstream infections, the role of linezolid in treating *Corynebacterium* bacteremia and endocarditis has gained increasing recognition. *C. striatum* has been reported to be multidrug-resistant, with reduced susceptibility to β-lactams, macrolides, and fluoroquinolones; however, it has been consistently reported to be sensitive to vancomycin.^3^,^5^,^8^ Linezolid has also proven to be effective; however, case reports of daptomycin resistance emerging during treatment have been reported, and it should be used with caution.^9^ In this regard, linezolid remains one of the few agents with retained in vitro activity.^6^,^7^,^10^ Favorable outcomes have been reported with linezolid in clinical case series and retrospective analyses, particularly when other agents were contraindicated or ineffective.^6^,^9^ Therefore, linezolid can be considered an appropriate treatment option in selected cases of confirmed *Corynebacterium* bacteremia or endocarditis, with susceptibility data supporting its use as in our case.

The clinical course of *C. striatum* endocarditis can be aggressive with complications such as abscess formation, valvular destruction and embolization.^3^,^7^ Our patient developed aortic root abscess, valve perforation and severe regurgitation with heart failure requiring urgent surgical intervention consistent with the severity observed in previous cases.^3^ An early transesophageal echocardiogram is essential to identify these complications early in the course of illness, and early intervention improves patient outcomes.

Published case series reports significant morbidity and mortality, particularly in elderly patients with comorbid conditions and prosthetic devices.^3^,^7^,^10^ However, favorable outcomes are achievable with early recognition, targeted antimicrobial therapy, and surgical intervention. In this case, the patient recovered from the infection and surgery, though he later died of intracerebral haemorrhage, which might be secondary to coagulopathy from anticoagulation, thrombocytopenia secondary to drugs, hypertensive bleed, or mycotic aneurysm rupture, although he was asymptomatic at the follow-up visit.

## CONCLUSION

*Corynebacterium* species can cause native-valve endocarditis, particularly in patients with predisposing cardiac lesions such as a bicuspid aortic valve. *C. striatum* bacteremia should not be disregarded in the presence of clinical findings; infection can be acquired after healthcare exposure, reinforcing the need for strict aseptic techniques. Infection can cause severe, destructive native valve disease in young adults. Early identification and timely surgical intervention can improve outcomes.

### Ethical considerations:

Written informed consent was secured from the patient, permitting the use of clinical information and imaging. An ethics review committee exemption was acquired for the publication of this case report. All personal identifiers have been removed to preserve anonymity, and confidentiality has been strictly maintained throughout the preparation of this manuscript.

### Authors’ Contribution:

**SA** conceived and designed the case report, provided clinical oversight of the case, contributed to drafting and revising the manuscript, and conducted the critical review and is responsible for the integrity of the work. **II**, **BM**, and **RM** contributed to data collection, drafting and revising the manuscript. All authors reviewed and approved the final version of the manuscript and agreed to be accountable for all aspects of the work.
